# Older Adults and New Technology: Mapping Review of the Factors Associated With Older Adults’ Intention to Adopt Digital Technologies

**DOI:** 10.2196/44564

**Published:** 2023-05-16

**Authors:** Tanja Schroeder, Laura Dodds, Andrew Georgiou, Heiko Gewald, Joyce Siette

**Affiliations:** 1 Centre for Health Systems and Safety Research Australian Institute of Health Innovation Macquarie University Macquarie Park Australia; 2 Centre for Research on Service Sciences (CROSS) Neu-Ulm University Neu-Ulm Germany; 3 The MARCS Institute for Brain, Behaviour and Development Western Sydney University Westmead Australia

**Keywords:** technology adoption, digital technology, older adults, seniors, intention to use digital technologies

## Abstract

**Background:**

Ongoing advancements in digital solutions support older adults’ healthy aging and well-being. However, a unified synthesis of sociodemographic, cognitive, attitudinal, emotional, and environmental factors that influence older adults’ intention to use these new digital technologies is still lacking. Understanding the salient factors that influence older adults’ intention to use digital technologies will help to ensure that technology is developed appropriately and contextually. This understanding is also likely to contribute to developing technology acceptance models specifically for the aging generation, by reorganizing principles and constructing objectivity criteria for future research studies.

**Objective:**

This review aims to identify the key factors associated with older adults’ intention to use digital technologies and to provide a comprehensive conceptual framework to describe the relationships between these key factors and older adults’ intention to use digital technologies.

**Methods:**

A mapping review was conducted using 9 databases from inception to November 2022. Articles were selected for review if they had an evaluative component of older adults’ intention to use digital technologies. Three researchers independently reviewed the articles and extracted the data. Data synthesis was performed via narrative review and quality appraisal was measured using 3 different tools based on each article’s study design.

**Results:**

We identified a total of 59 articles investigating older adults’ intention to use digital technologies. The majority (40/59, 68%) of articles did not use an existing framework or model for technology acceptance. Studies mostly adopted a quantitative research design (27/59, 46%). We found 119 unique factors reported to influence older adults’ intention to use digital technologies. These were categorized into 6 distinct themes: Demographics and Health Status, Emotional Awareness and Needs, Knowledge and Perception, Motivation, Social Influencers, and Technology Functional Features.

**Conclusions:**

Given the importance of global demographic change toward an aging society, there is surprisingly limited research on the factors that influence older adults’ intention to use digital technologies. Our identification of the key factors across different types of digital technology and models supports the future integration of a comprehensive perspective encompassing environmental, psychological, and social determinants for older adults’ intention to use digital technologies.

## Introduction

Technological innovation and the constantly increasing use of the internet are creating unique opportunities to assist older adults’ (here defined as persons with a physical age of 65 years and above) health and well-being [1]. There is growing evidence of the benefits that older adults experience when they use digital technologies. These include improvements in their cognitive, social, and emotional well-being [2]. Technologies such as computers, the internet, and mobile phone apps have been found to be effective tools in managing health conditions and supporting well-being [3]. Although older adults’ adoption rates of technology have traditionally been low, they are nowadays increasing and the gap toward the younger generations is closing significantly [4]. In this era of increased global aging, the World Health Organization estimates that by 2030, 1 in 6 people in the world will be aged 60 years or over [5], and that it is necessary that technological developments become more age-friendly and usable by older adults. However, the factors influencing older adults’ intention to use digital technologies are not yet fully understood.

The information systems discipline has developed various models of technology acceptance to understand the factors leading to the acceptance, adoption, use, and continuous use of technology. Among the most widely used theoretical frameworks are the Technology Acceptance Model (TAM) [6] and the Unified Theory of Acceptance and Use of Technology (UTAUT) [7], which have been developed based on broadly defined adult populations with expansive age brackets [8].

Formulated by Davis [6], the TAM describes individuals’ *acceptance* of technologies [9] and has been applied to a wide variety of contexts including the health care sector [10]. The TAM suggests that perceived usefulness (ie, whether the users perceive the technology as helpful to achieve the intended purpose) and perceived ease of use (ie, whether the users perceive the technology to be easy to use for them) explain an individuals’ intention to use digital technology [6,11]. Based on the Theory of Reasoned Action [12], intention is regarded as a powerful predictor of actual use. However, the main points of criticism for the TAM are that its measurement relies on self-reported perceptions and that the dependent variable is behavioral intention, not the actual use of a technology [13]. Furthermore, the model does not consider factors including age and education, external variables which could influence willingness to use technology [14]. As such, more recent model developments have taken place.

The UTAUT model [7], developed by Venkatesh et al [7], combines and integrates 8 theories to explain human behavior with respect to technology adoption. It identifies 4 major constructs (performance expectancy, effort expectancy, social influence, and facilitating conditions), along with 4 moderators (age, gender, experience, and voluntariness) to predict individuals’ use of technology [7,15]. In 2012, the UTAUT model was further developed into the UTAUT 2 model by Venkatesh et al [8]. The authors extracted factors for the consumer context and extended it by incorporating another 3 factors, namely, hedonic motivation, price value, and habit, which improved the prediction of behavioral intention to use behavior. The UTAUT 2 includes 3 moderators: age, gender, and experience [8]. The TAM and UTAUT received enormous attention in academia and practice, and probably belong to the most tested, adapted, and extended models in information systems research. However, both models share the same weakness that they were not developed with consideration of different application areas, which can be beneficial or detrimental. Furthermore, they do not incorporate the fact that technology acceptance may change over time [11].

In 2000, the original TAM was expanded by Venkatesh and Davis [16] with some elements and republished as TAM2, with further revisions made in 2008 to create TAM3 [17]. In TAM2, the input variables were differentiated into the groups of social influence and cognitive processes. TAM3 is based on the acceptance variables of the original TAM (ie, perceived usefulness, perceived ease of use, and intention to use) and TAM2 (ie, experience, voluntariness, subjective norm, image, workplace relevance, quality of outcome, and presentable results). This model is supplemented by the subcategories anchor and adjustment, which include computer self-efficacy, perception of external control, computer anxiety and computer playfulness, as well as perceived enjoyment and objective usability. The Senior Technology Acceptance Model [18] also describes a further development of the TAM and captures the context of the older mobile phone user. Here, intention to use is primarily determined by perceived usefulness and social influence (ie, children urging their parents to use the phone). The variables are social influence, intention to use, perceived usefulness, facilitating conditions, experimentation and exploration, confirmed usefulness, ease of learning and use, and actual use.

However, despite these adjustments, within gerontology and aging research fields, a widespread deficiency of the existing technology acceptance models is the neglect of biophysical factors (eg, cognitive and physical decline) and psychosocial factors (eg, social isolation and fear of illness) [19]. As such, extant theoretical models of technology acceptance are not fully applicable to members of the aging population [20].

This systematic mapping review is set to capture the diverse literature available on this topic and provide in-depth insights into overarching concepts to further advance this field. Furthermore, a framework that incorporates the most up-to-date evidence of how the key factors interact and their impact on older adults’ behavioral intentions is yet to be produced. By tracking the flow of information through publications using a mapping review, linkages between core concepts related to the intended use of technology across disciplines can be identified [21,22].

This mapping review thus provides a synthesis of current research on older adults’ intention to use digital technology and the corresponding salient factors. Our objective was 2-fold: (1) to identify the key factors associated with older adults’ intention to use digital technologies, and (2) to provide a comprehensive conceptual framework to describe the relationships between these key factors and older adults’ intention to use digital technologies. These findings will provide directions for further research addressing the specific user group of older adults.

## Methods

### Study Design

**Figure 1:**
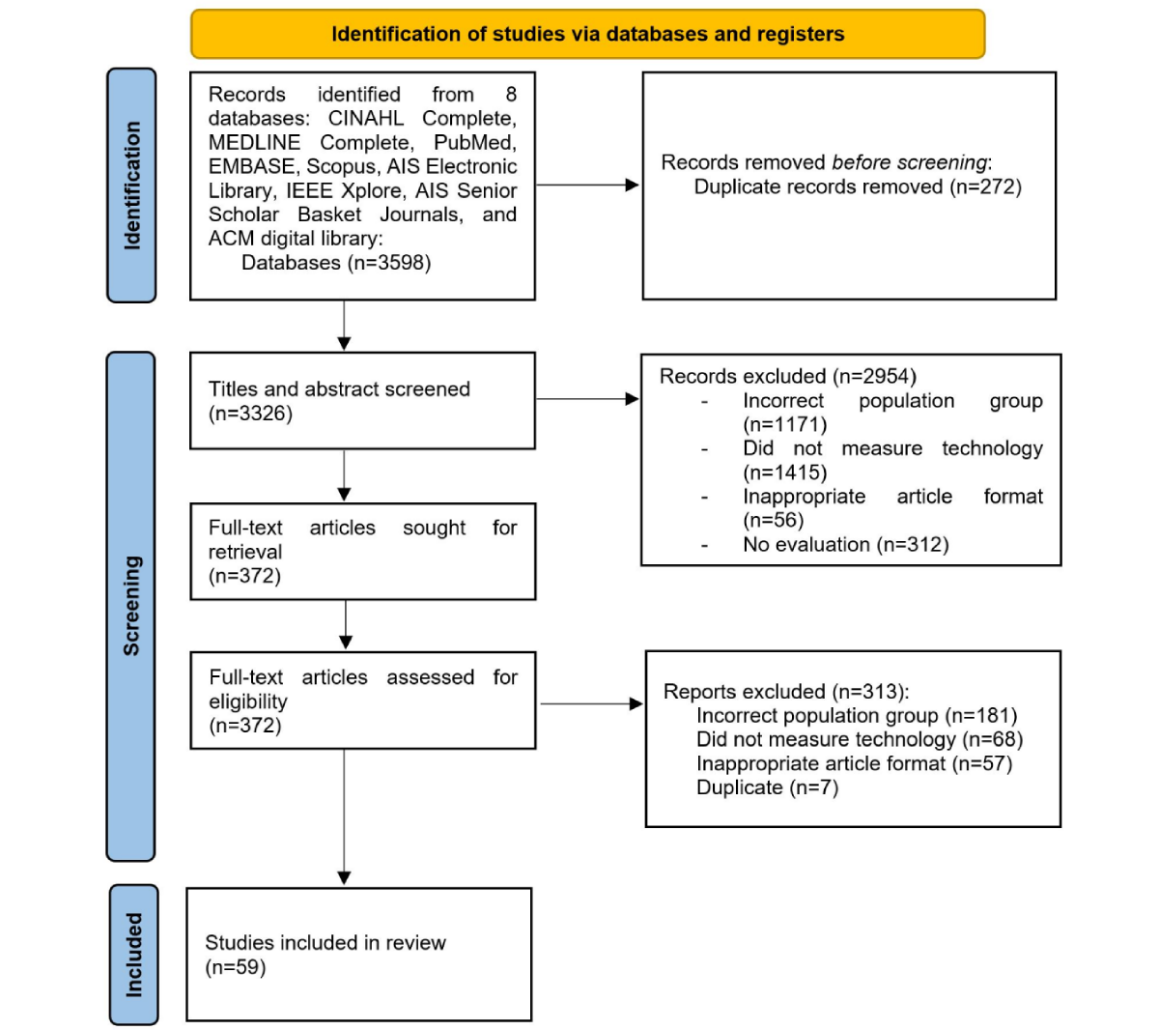
PRISMA (Preferred Reporting Items for Systematic Reviews and Meta-Analyses) flowchart.

A systematic mapping review was used to identify the published original articles related to intention to use digital technology by older adults. The review protocol was registered with PROSPERO (International Prospective Register of Systematic Reviews; registration number CRD42022329705) and the selection process is outlined in Figure 1.

### Search Strategy and Study Selection

A search was conducted from inception to November 15, 2022, in 9 databases including CINAHL Complete, MEDLINE Complete, PubMed, Embase, and Scopus, as well as in 3 information systems–focused databases, AIS Electronic Library, IEEE Xplore, and ACM Digital Library, and in the journals of the AIS Senior Scholar Basket. Broad keywords used were the following combination of 4 groups of keywords: older adults (eg, aged, older, senior, elderly) AND technology factor (eg, acceptance, adoption, use, adherence, rejection) AND influence (eg, behaviour, psychology, motivation) AND technology type (eg, technology, computers, eHealth, system, assistive technology, robotics, smart home, gerontechnology, telemonitoring). The full search strategy was updated on November 15, 2022 and is provided in Multimedia Appendix 1.

### Inclusion Criteria

Original and peer-reviewed journal publications and conference proceedings written in English and using qualitative, quantitative, or mixed methods research were included. Studies had to include participants who were older adults (aged 65 years or older) and had an evaluative component of either demographic, cognitive, physical, or emotional factors that influenced the intention to use or actual use of digital technology.

All titles and abstracts of the identified studies were subsequently screened for eligibility by 3 reviewers (JS, LD, and TS) independently, applying the following inclusion criteria: (1) original and peer-reviewed research written in English with either a qualitative, quantitative, or mixed methods approach; (2) participants were older adults aged 65 years or more; and (3) research was aimed at investigating factors that influence the intention to use or the actual use of digital technologies for older adults. The 3 researchers then conferred to resolve any discrepancies on eligibility, and if an agreement could not be made, a fourth reviewer (AG) was consulted. The full text of the remaining studies was then checked and those that did not meet all inclusion criteria were excluded. The references of the selected studies were hand searched for other potential studies (snowballing method; Figure 1).

### Assessment of Methodological Quality

Methodological quality was assessed using the Critical Appraisal Skills Program, the Cochrane Risk of Bias, and the Mixed Methods Appraisal Tool (MMAT) [23] which, in addition to specific criteria for qualitative and quantitative research, also contains specific criteria on the relevance of the use of a mixed methods design and the integration of different types of results. The researchers assessed the methodological quality of all included studies independently, followed by a discussion of their findings to determine the final rating for each study. It was decided not to exclude articles based on quality assessment because there is little empirical evidence on which to base exclusion decisions in mixed studies systematic reviews [23-25]. Instead, it was decided to report on the quality of the reviewed articles and to apply independent triangulation: at least two quality criteria had to be present in studies to be included in the results.

### Data Extraction and Synthesis

As included articles ranged from qualitative to quantitative methods (or a combination of both), data extraction forms for both design types were designed. For qualitative articles and qualitative information from mixed methods articles, acceptance factors were coded and entered. For quantitative articles and quantitative information from mixed methods articles, variable/factor name and level of significance were entered into the data extraction form. Two authors (TS and JS) reached consensus on the data extraction form for each article.

Thematic synthesis [25] was used to synthesize qualitative data on technology use and intention to use factors. Multiple sessions in the research team were then held to group factors derived from articles into descriptive themes for acceptance in post-technology implementation. JS, LD, and TS each created a conceptual model of the relationships between themes, and through iterative discussions developed 1 final model. Factors derived from qualitative articles and mixed methods articles were compared with factors identified in quantitative articles. This allowed us to highlight which factors were statistically tested in quantitative research. Quantitative articles were summarized thematically. For this purpose, all factors examined were first extracted and compiled. We summarized thematically related factors into individual group categories to create a better overview. Cluster analysis was used as an explorative method to establish the group categories and to classify the individual factors by purpose and type. Subsequently, we also added the factors examined in the qualitative articles.

## Results

### Overview

The study characteristics of the 59 articles analyzed are shown in Table 1. Most articles (36/59, 61%) were written in the last 5 years, published in the United States (25/59, 42%), and conducted in a community-dwelling setting (23/59, 39%), with 39,153 older adults sampled (sample size ranged from 5 to 14,798).

**Table 1:** Summary of the included articles.

Authors	Country	Study design	Setting	Sample size, n	Sample mean age (years)	Proportion of female, %	Technology type	Technology subtype	Purpose				Model used
									Health and safety	Social interaction	Independence		
Franks and Beckmann [26]	Canada	MM^a^	Other and home care	100	77.5	60	Remote or assistive care technologies	Hearing aids	✓	—^b^	✓	—	
Billipp Heyn [27]	United States	E^c^	Home care	40	73	82	Everyday technologies	Computer	—	✓	✓	—	
Jimison et al [28]	United States	E	Home care	15	79.5	—	Everyday technologies	A computer game	—	—	✓	—	
Kurt et al [29]	United States	Q^d^	Other	240	77.5	—	Everyday technologies	Client-server computer application	—	—	✓	—	
Horowitz et al [30]	United States	Q	Community center	438	80.4	—	Remote or assistive care technologies	Optical and adaptive device	✓	—	✓	—	
Courtney et al [31]	United States	Q	Community dwelling	14	77.5	—	Remote or assistive care technologies	Smart home device	✓	—	✓	Health Belief Model	
Demiris et al [32]	United States	O^e^	Home care	9	82.5	—	Remote or assistive care technologies	Smart home device	✓	—	✓	—	
Chu et al [33]	United States	Q	Community center	137	77.5	72	Everyday technologies	Computer	—	✓	✓	Bandura’s Self-Efficacy Theory	
Beer and Takayama [34]	United States	E	Community dwelling	12	73.4	58.30	Everyday technologies	Mobile	—	—	✓	—	
Chudyk et al [35]	Canada	Q	Primary care	28	71	50	Remote or assistive care technologies	Monitoring device	✓	—	—	—	
Cornejo [36]	Mexico	E	Community dwelling	12	86.5	—	Everyday technologies	Sensors	—	—	✓	—	
Aloulou et al [37]	Singapore	S^f^	Long-term care	8	85	100	Remote or assistive care technologies	Sensors	✓	—	✓	—	
Choi and Dinitto Diana [38]	United States	Q	Community dwelling	6680	77.5	56	Everyday technologies	Internet	—	✓	✓	—	
Demiris et al [39]	United States	E	Home care	27	86	27	Remote or assistive care technologies	A health management platform (CogniFit, GAITRite, and telehealth kiosk)	✓	—	—	Hoyman’s Wellness Model	
Baig et al [40]	New Zealand	E	Primary care	30	82.4	43	Remote or assistive care technologies	Monitoring system	✓	—	✓	—	
Colombo et al [41]	Italy	Q	Community dwelling	900	68.5	—	Everyday technologies	Smartphone, tablet, computer	—	✓	✓	—	
Gellis et al [42]	United States	R^g^	Primary care	102	77.5	—	Remote or assistive care technologies	Telehealth	✓	✓	—	—	
Aponte and Nokes Kathleen [43]	United States	Q and S	Community center	20	74	50	Everyday technologies	Internet	—	✓	✓	—	
Jimison et al [44]	United States	O	Community dwelling	6	82.5	67	Everyday technologies	Video	—	—	✓	Trans-Theoretical Model of Behavioral Change	
Neves et al [45]	Canada	MM	Long-term care	5	87.2	60	Everyday technologies	Mobile	—	✓	—	—	
Chopik [46]	United States	Q and S	Community dwelling	591	68	56	Everyday technologies	Internet	—	✓	✓	—	
Dupuy et al [47]	France	E	Community dwelling	34	85	—	Remote or assistive care technologies	Assistive device	✓	—	✓	Self-Determination Theory	
Cajita et al [48]	United States	S	Primary care	129	71.3	26	Everyday technologies	Mobile	—	✓	✓	TAM^h^	
Chang et al [49]	Taiwan	Q	Primary care	18	77.6	67	Remote or assistive care technologies	Telehealth	✓	✓	—	TAM2^i^	
Chung et al [50]	United States	Q	Community dwelling	21	69.6	—	Remote or assistive care technologies	Monitoring systems	✓	—	—	TAM	
Djabelkhir et al [51]	France	R	Primary care	20	76.7	45	Everyday technologies	Computerized cognitive stimulation	—	✓	✓	—	
Irizarry et al [52]	United States	MM	Community dwelling	100	77.5	—	Remote or assistive care technologies	Patient portal	✓	—	—	—	
Cajita et al [53]	United States	Q	Primary care	10	69.5	40	Remote or assistive care technologies	mHealth^j^	✓	—	—	TAM	
Dugas et al [54]	United States	E	Primary care	27	67.6	—	Everyday technologies	A gamified mHealth Tool (DiaSocial)	✓	✓	—	Regulatory Mode Theory	
Holgersson and Söderström [55]	Sweden	Q	Community dwelling	293	77.5	—	Everyday technologies	E-services	—	—	✓	—	
Khosla et al [56]	Australia	MM	Home care	5	80	—	Remote or assistive care technologies	Social robot	✓	✓	✓	TAM	
Mohlman and Basch [57]	United States	S	Community dwelling	85	77.2	60	Everyday technologies	—	—	✓	✓	—	
Kim et al [58]	Republic of Korea	S	Primary care	21	72	81	Everyday technologies	Mobile app	✓	✓	✓	—	
Woo et al [59]	United States	Q	Home care	20	72.6	55	Remote or assistive care technologies	Telehealth	✓	✓	—	UTAUT^k^	
Ali et al [60]	Australia	Q	Other/undefined^l^	14,798	—	52	Remote or assistive care technologies	E-services	✓	✓	—	—	
Bernstein et al [61]	United States	S	Community dwelling	60	73.4	27	Everyday technologies	Computer and apps	—	✓	✓	—	
Bevilacqua et al [62]	Italy	MM	Other/undefined	35	74	63	Remote or assistive care technologies	Robot	✓	✓	✓	—	
Cohen-Mansfield et al [63]	Israel	Q	Home care	105	74	85	Everyday technologies	A gamified web-based activity program	—	—	✓	—	
Doyle et al [64]	Ireland and Belgium	S	Home care	120	74.2	40	Remote or assistive care technologies	A digital health platform (ProACT)	✓	—	—	—	
Ghorayeb et al [65]	United Kingdom	Q	Home care	13	77	54	Remote or assistive care technologies	Smart home devices	✓	—	✓	—	
Harris et al [66]	United States	S	Community dwelling	70	71.1	73	Everyday technologies	Mobile	✓	✓	✓	—	
Kalicki et al [67]	United States	S	Community dwelling	873	82.7	75	Remote or assistive care technologies	Telehealth	✓	✓	—	—	
Klaver et al [68]	The Netherlands	S	Primary care	463	74	49	Remote or assistive care technologies	mHealth apps	✓	—	✓	TAM/UTAUT	
Li et al [69]	United States	S	Primary care	2909	77.2	55	Everyday technologies	Information and communications technology	—	✓	✓	—	
Marklund et al [70]	Sweden	Q	Primary care	16	72	75	Remote or assistive care technologies	eHealth	✓	—	—	Fogg Behavior Model	
Nebeker and Zlatar [71]	United States	R	Other/undefined	41	71.6	76	Remote or assistive care technologies	mHealth	✓	—	✓	—	
Pang et al [72]	Canada	MM	Community dwelling	42/27	69	43	Everyday technologies	Learning video prototype	—	—	✓	—	
Yachin and Nimrod [73]	Canada, Colombia, Israel, Italy, Peru, Romania, and Spain	S	Other/undefined^l^	184	71	100	Everyday technologies	Information and communications technology	—	✓	✓	—	
Blavette et al [74]	France	S	Primary care	45	82	65	Remote or assistive care technologies	Robot	✓	✓	—	—	
Choi et al [75]	United States	MM	Community dwelling	3257	—	56	Remote or assistive care technologies	Telehealth	✓	✓	—	Healthcare Utilization Model	
Coley et al [76]	France	R	Community dwelling	2724	69	48	Remote or assistive care technologies	Internet-based intervention	✓	✓	✓	—	
Domingos et al [77]	Portugal	S	Community dwelling	110	69	66	Everyday technologies	Activity tracker	—	—	✓	TAM3^m^	
Gomez et al [78]	United Kingdom	O	Community dwelling	44	72	—	Remote or assistive care technologies	Hearing aid	✓	—	—	—	
Krutter et al [79]	Germany	MM	Home care	20	65	—	Remote or assistive care technologies	Virtual avatar	✓	—	—	UTAUT	
Özsungur [80]	Turkey	S	Home care	687	73	40	Everyday technologies	Online services and apps	✓	✓	✓	UTAUT 2^n^	
Qin [81]	United States	S	Community dwelling	1769	—	55	Remote or assistive care technologies	Telehealth	✓	✓	—	—	
Smrke et al [82]	Slovenia	S	Home care	185	81	66	Remote or assistive care technologies	Robot	—	✓	✓	—	
Vailati Riboni et al [83]	Italy	Q	Community dwelling	68	72	71	Everyday technologies	App	✓	—	✓	—	
Van Elburg et al [84]	The Netherlands	S	Community dwelling	360	75	57	Remote or assistive care technologies	mHealth apps	✓	—	✓	TAM^o^	

^a^MM: mixed methods study.

^b^Data not available.

^c^E: experimental study.

^d^Q: qualitative study.

^e^O: observation study.

^f^S: survey study.

^g^R: randomized control trial.

^h^TAM: Technology Acceptance Model.

^i^TAM2: Technology Acceptance Model 2.

^j^mHealth: mobile health.

^k^UTAUT: Unified Theory of Acceptance and Use of Technology Model.

^l^Others/undefined: The provided information does not fit in the settings (eg, free-living environment, Facebook group) or the information was not provided.

^m^TAM3: Technology Acceptance Model 3.

^n^UTAUT 2: Unified Theory of Acceptance and Use of Technology Model 2.

^o^Includes variables of TAM2 and the Senior Technology Acceptance Model, but we focused on the dominant model TAM.

Different types of technology were examined, ranging from mobile technologies and television-based to assistive technologies, with the most common technology type being technology devices. Based on the scope of functionalities, 2 main categories of technology were summarized [85]: (1) everyday technologies (including hardware devices, such as computers, smartwatches, tablets, computers, and services such as gaming, apps, and other technologies used to support daily living); and (2) remote or assistive care technologies, which were those that use information communication technology devices and networks to deliver health and social care technology. Remote or assistive care technologies were most commonly reported (32/59, 54%).

The purpose of technology was also defined. Technology that supports older adults’ independence through activities of daily living or instrumental activities of daily living was the most common (40/59, 68%), followed by technology that aims to enhance safety (eg, such as monitoring or assistive technology; 36/59, 61%).

In a 2008 study [31], a model was used for the first time as a theoretical basis. Subsequently, studies from 2015 onward have used models for their studies. The studies did not show a trend in terms of setting, study design, technology type, and model use. Most study designs used were quantitative (27/59, 46%), followed by qualitative (21/59, 36%) and mixed methods (11/59, 19%).

### Use of Theoretical Models

Only one-third (19/59) of the articles used 1 of the available theoretical models to explain older adults’ intention to use digital technology. The remaining articles provided descriptive evidence to support, formulate, or extend a theoretical model. Table 2 provides an overview of the theories used, the research fields they derive from, and the articles that applied them to their research.

A classification of the 12 models based on academic discipline of their origin and the constructs applied by the corresponding research article is provided in Table 3. Table 4 presents a summary of the used constructs grouped into categories in articles that used and did not use a theoretical basis.

**Table 2:** Use of theoretical models.

Model used	Research field	Total	Article(s)
Bandura’s Self-Efficacy Theory	Psychology	1	[33]
Health Belief Model	Health	1	[31]
Hoyman’s Wellness Model	Health	1	[39]
Healthcare Utilization Model	Health	1	[75]
Regulatory Mode Theory	Sociopsychology	1	[54]
Self-Determination Theory	Sociopsychology	1	[47]
Trans-Theoretical Model of Behavioral Change	Sociopsychology	1	[44]
Fogg Behavior Model	Sociopsychology	1	[70]
Technology Acceptance Model	Information systems	6	[48,50,53,56,68,84]
Technology Acceptance Model 2	Information systems	1	[49]
Technology Acceptance Model 3	Information systems	1	[77]
Unified Theory of Acceptance and Use of Technology	Information systems	3	[59,68,79]
Unified Theory of Acceptance and Use of Technology 2	Information systems	1	[80]
Articles using models	N/A^a^	19	N/A
No model used	N/A	40	N/A
Total articles	N/A	59	N/A

^a^N/A: not applicable.

**Table 3:** Classification of the used models.

Research field	Model	Constructs
Information systems	Technology Acceptance Model Technology Acceptance Model 2 Technology Acceptance Model 3 Unified Theory of Acceptance and Use of Technology Unified Theory of Acceptance and Use of Technology 2	Computer anxiety; Computer playfulness; Computer self-efficacy; Confirmed usefulness; Ease of learning and use; Experimentation and exploration; Facilitating conditions; Habit; Hedonic motivation; Image; Job relevance; Objective usability; output quality; Perceived ease of use; Perceived enjoyment; Perceived usefulness; Perception of external control; Price value; Result demonstrability; Social influence; Subjective norm; User context
Health	Hoyman’s Wellness Model Health Belief Model Healthcare Utilization Model	Enabling factors (family support, access to health insurance); Mental and cognitive health; Perceived barriers; Perceived benefits; Perceived or actual needs; Perceived severity; Perceived susceptibility; Physical well-being; Predisposing factors (demographics); Social well-being; Spiritual well-being
Sociopsychology	Fogg Behavior Model Trans-Theoretical Model of Behavioral Change Self-Determination Theory Regulatory Mode Theory	Ability; Autonomy; Competence; Consciousness raising; Counter-conditions; Dramatic relief; Environmental reevaluation; Helping relationships; Locomotion and assessment; Motivation; Promoting value; Prompts; Reinforcement management; Relatedness; Self-liberation; Self-reevaluation; Social liberation; Stimulus control
Psychology	Bandura’s Self-Efficacy Theory	Emotional and physiological states; Performance outcomes (mastery experiences); Social persuasion; Social role models (vicarious experiences)

**Table 4:** Summary of constructs grouped into categories^a^.

Demographics and Health Status		Emotional Awareness and Needs	Knowledge, Competence, and Perception			Motivation		Social Influencers		Functionals features	
Age BMI Chronic disease Cognitive impairment Cognitive performance Depression Employer Functional disability Gender Hearing ability Linguistic problems Marital status Origin Sensory perception *Income* *Existing disease* *Medical history* *Loneliness* *Physical activity* *Presence of dementia* *Professional education* *Quality of life* *Religion* *Cultural issues* *Physical weakness*	Anxiety Application design Self-determination Self-efficacy Self-esteem *Confidence* *Confidentiality* *Emotional needs* *Fear* *Feelings* *Intensity* *Mood* *Need for emotional support* *Pain* *Patience* *Perceived loneliness* *Resistance to change* *Stigma consciousness*			(Adequate) computer training Education (Previous) experience Lack of knowledge Perceived ease of use Perceived privacy Perceived risk expectancy Perceived usefulness *Beliefs* *Digital literacy* *Empowerment* *Health literacy* *(Medication) adherence* *Memory* *Misuse/overuse* *Patients’ choices* *Perceived access barriers* *Personal innovativeness* *Previous experience* *Privacy concerns* *Satisfaction* *Self-management* *Trust in provider* *Trust in system* *Learning a new technology* *Level of assessment/capability*	Benefits Habit Perceived threat *Attitude* *Awareness* *Curiosity* *Equipment needs* *Perception of need* *Regularity* *Rewards* *Lack of need for technology* *Willingness to learn* *Reduced in-person health care* *Engagement*		Social influence Subjective norm *Interpersonal relationships* *Learn with help* *Social capital* *Social isolation* *Social participation* *Social perceptions* *Personal influence* *Support* *Social relationships* *Physicians’ recommendation*		Cost External influence Facilitating conditions Usability User friendliness Ease of use Presence of useful features *Accessibility* *Assurances of technology* *Compatibility* *Feasibility* *Impressions of technology* *Inconvenience to wear* *Information quality* *Portal use* *Self-perceived effectiveness of use* *Service ability* *Service quality* *System quality* *Utility of technology for health* *Active versus passive communication* *Ambiguous affordances* *Poorly designed interface*		

^a^Constructs not used in the models are given in italics.

The information systems field developed the TAM (n=6), TAM2 (n=1), TAM3 (n=1), UTAUT (n=3), and UTAUT 2 (n=1). These models focused on core constructs of perceived usefulness and perceived ease of use, attitude toward using, and intention to use the system (eg, TAM). The UTAUT also included the construct social influence and facilitating conditions. Both the TAM and the UTAUT were further developed, including TAM2 (n=1) and TAM3 (n=1), which highlighted trust and perceived risk on system use, and UTAUT 2 (n=1), which incorporated 3 other constructs including hedonistic motivation (eg, the pleasure of using a technology), price-performance ratio, and habits.

Three models had their origin in health-related research. Both the Health Belief Model [86,87] and Hoyman’s Wellness Model [88] explored the multidimensional unit of health and wellness by emphasizing human health needs holistically within their environment by addressing 4 dimensions. Finally, Anderson’s Healthcare Utilization Model [89] aimed to understand how and why people use health care services, assess inequalities in accessing health services, and aid in the creation of policies that will allow for equitable access to care. To predict or explain one’s use of health care services, the model particularly focused on an individual’s predisposition to use acute health care services, enabling factors that facilitate use and one’s perceived or influenced need for care.

From the sociopsychological domain, the Trans-Theoretical Model of Behavioral Change [90] described an integrative theory of therapy that assesses an individual’s readiness to act on a new healthier behavior, providing strategies or processes of change to guide the individual. The model is composed of constructs such as stages of change, processes of change, levels of change, self-efficacy, and decisional balance. The Regulatory Mode Theory [91] described how people approach situations to achieve their goals. Similarly, the Fogg Behavior Model [92] labeled main motivators (motivation), factors of simplicity (ability), and the types of prompts for goal acquisition constructs. The Self-Determination Theory [93] predicted health-related behaviors; however, the Health Belief Model focuses on the behavioral determinants influencing uptake of health-related behaviors, while the Self-Determination Theory explains behavioral motivation as dependent on whether basic psychological needs for competence, social inclusion, and autonomy can be satisfied.

From the research field psychology, the Self-Efficacy Theory [94] explained how well a person that can cope with the particular situation is dependent on the skills they have and the circumstances they face.

### Technology Types and Purpose

Articles showed that older adults had varying acceptance levels to different forms of technology. Two different types of digital technologies were found that focused on (1) everyday technologies or (2) remote or assistive care technologies (Table 5). We further assigned these types by purpose using categories identified by Peek et al [11] that explored how the technology type supported the users’ health and safety, social interaction, or independence.

**Table 5:** A summary of models identified through technology type and purpose.

Model used			Article(s)		Technology type					Technology purpose				
					Everyday technologies		Remote or assistive care technologies	Health and safety				Social interaction		Independence
Fogg Behavior Model			[70]		—^a^		1	✓				—		—
Health Belief Model			[31]		—		1	✓				—		✓
Hoyman’s Wellness Model			[39]		—		1	✓				—		—
Healthcare Utilization Model			[75]		—		1	✓				✓		—
Regulatory Mode Theory			[54]		1		—	✓				✓		—
Self-Determination Theory			[47]		—		1	✓				—		✓
Bandura’s Self-Efficacy Theory			[33]		—		1	—				✓		✓
Technology Acceptance Model			[48,50,53,56,68,84]		1		5	✓				✓		✓
Technology Acceptance Model 2			[49]		—		1	✓				✓		—
Technology Acceptance Model 3			[77]		1		—	—				—		✓
Trans-Theoretical Model of Behavioral Change			[44]		1		—	—				—		✓
Unified Theory of Acceptance and Use of Technology			[59,68,79]		—		3	✓				✓		✓
Unified Theory of Acceptance and Use of Technology 2			[80]		1		—	✓				✓		✓

^a^Data not available.

Models were more commonly applied to remote or assistive care technologies (15/19, 79%), with the TAM and UTAUT models identified in more than 1 article [68]. Other models were used less frequently. Similarly, only the TAM, UTAUT, and UTAUT 2 models were found in articles exploring all 3 technology purposes (eg, [59,68,79,80]).

### Technology Types by Setting

The setting in which the research participants reside is expected to have a major influence on their intention to use digital technology. Thus, we analyzed which technology types were studied in what setting. Table 6 provides the corresponding overview.

The setting most studied was community dwelling (24/59, 41%), followed by primary care (13/59, 22%) and home care (12/59, 20%). Community dwelling refers to older people who live independently within the community. Primary care refers to health services that include a range of preventive, wellness, and treatment measures for common diseases. Primary care providers include doctors, nurses, nurse practitioners, and health care professionals. Home care refers to the nursing and domestic care of people in need of care outside of partial or full inpatient facilities in their home environment. Overall, technology types were relatively distributed between settings, although home care and primary settings tended to have more remote or assistive care technology types, while everyday technologies were more likely to be found in community dwelling and community centers.

**Table 6:** Technology types studied in different settings by articles.

Setting	Technology type			Total (N=59)
	Everyday technology (n=27), n (%)	Remote or assistive care technology (n=32), n (%)		
Community center	2 (67)	1 (33)	3	
Community dwelling	13 (57)	10 (43)	23	
Home care	4 (33)	8 (67)	12	
Long-term care	1 (50)	1 (50)	2	
Primary care	5 (38)	8 (62)	13	
Other/undefined	2 (33)	4 (67)	6	

### Factors Contributing to Older Adults’ Intention to Use Digital Technology

#### Overview of Categories

Table 7 provides a list of the constructs used to explain and predict older adults’ intention to adopt technology. To provide a meaningful analysis of the numerous constructs we grouped them into 6 categories: Demographics and Health Status, Emotional Awareness and Needs, Knowledge and Perception, Motivation, Social Influencers, and Technology Functional Features (for further information on these constructs, see Table 4 and Multimedia Appendix 1). Table 7 shows the number of articles that applied a specific category to a certain technology type.

**Table 7:** Articles reporting an intention to use technology by category and technology type.

Category	Articles that applied this category (N=59), n (%)	Technology type	
		Everyday technologies, n (%)	Remote or assistive care technologies, n (%)
Demographics and Health Status	37 (63)	15 (41)	22 (59)
Emotional Awareness and Needs	21 (36)	11 (52)	10 (48)
Knowledge, Competence, and Perception	40 (68)	15 (38)	25 (62)
Motivation	24 (41)	12 (50)	12 (50)
Social Influencers	22 (37)	11 (50)	11 (50)
Functional Features	36 (61)	16 (44)	20 (56)

The most commonly identified category was *Knowledge, Competence, and Perception* (40/59, 68%), which explored how education, privacy concerns, trust in the provider, and health literacy influenced behavioral intention. This was followed by *Demographics and Health Status* (37/59, 63%), which explored the concepts of age, gender, origin, as well as health status and medical history. *Functional Features* was also frequently described (36/59, 61%) and considered how technical capabilities and concepts such as better information quality, interoperability, and service and system quality affected behavioral intention.

Overall, technology types were relatively distributed between the categories. Remote or assistive care technologies were observed more frequently in the categories of Knowledge, Competence, and Perception (25/40, 63%) and Demographics and Health Status (22/37, 59%). More detailed descriptions of the categories and their direction of influence are described in the following sections.

#### Demographics and Health Status

Health limitations such as physical inability, presence of dementia, functional disabilities, and other diseases had a negative impact on the intention to use digital technology. By contrast, marital status, healthy BMI, and higher income were linked with higher behavioral intention. These individual factors as well as age were also considered moderators in other studies.

#### Emotional Awareness and Needs

This category explored the concepts of anxiety, fear, self-determination, self-efficacy, and pain, and was reported across the majority of technology types. Older adults' self-determination, stigma-consciousness, and self-efficacy were linked to increased behavioral intention. By contrast, resistance to change, fear, and anxiety were found to negatively impact on the intention to use digital technology.

#### Knowledge, Competence, and Perception

Older adults that had high perceived risk during technology use, privacy concerns, or lack of trust in the provider and the system, poor prior experience, low knowledge, and low health literacy had lower behavioral intentions. By contrast, if older adults could make their own choices, had prior computer training, had positive expectancy, and had prior strong satisfaction, they would be more likely to use the technology.

#### Motivation

This category reflected users’ motives and intention. Concepts included perceived need for the technology, available rewards, attitudes, goals, and habits. Individuals were more likely to use technology when they set health goals and perceived a need to use a technology to support their goal.

#### Social Influencers

This category considered social determinants of general social pressure on a person to engage in a particular behavior and included factors such as isolation, participation, social capital, and network support. The presence of positive social support supported behavioral intention in 11 articles in each technology type.

#### Functional Features

This category described the technical characteristics of a technology that fulfill a specific function. This included factors such as accessibility, usability, cost, system and service quality, and design. Users who could operate a technology better found the design more appealing, and were technically more capable and more likely to adopt the technology.

#### Conceptual Framework

**Figure 2:**
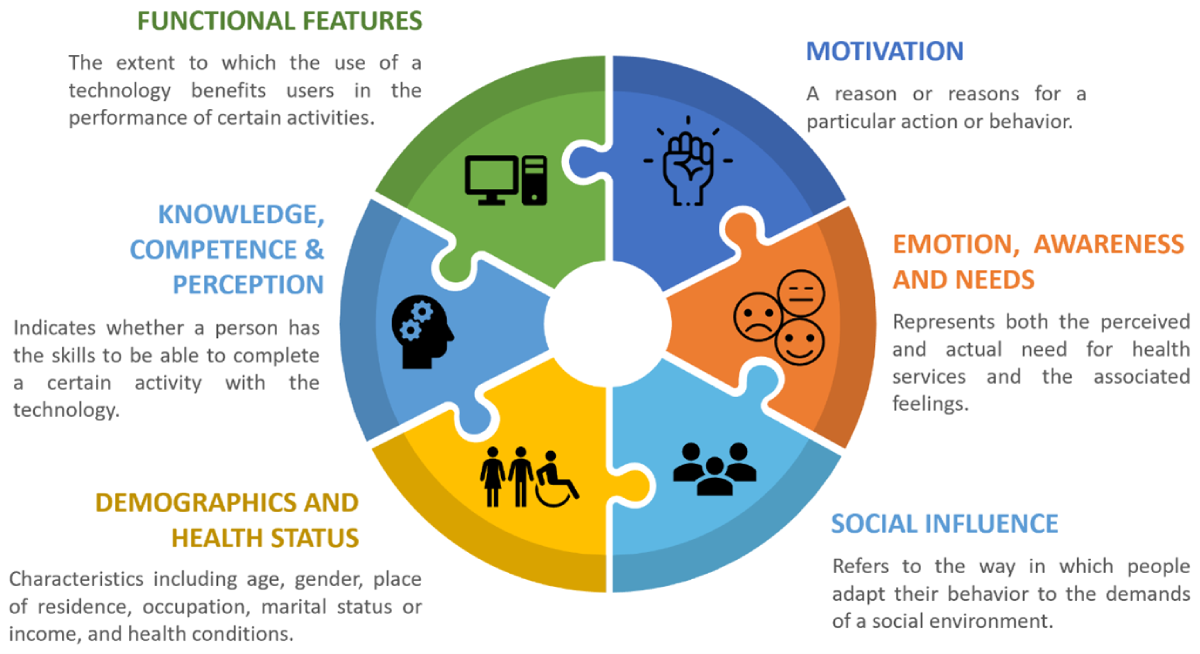
A collective framework of influencing factors.

Based on the information derived from our review, there were 6 clusters of influencing factors from sociopsychological, psychological, and health information fields, which describe older adults’ intention to use digital technology. We have combined these to present a unified perspective, which provides a more comprehensive and collective view of the factors influencing older adults’ behavioral intention across multiple disciplines (Figure 2). This framework emphasizes the interconnected role of the 6 constructs that influence older adults’ intention to use technology across multiple technology types.

#### Quality Appraisal

Overall, the quality of the studies was strong for qualitative and quantitative research designs, and moderate for mixed method designs (Multimedia Appendix 1). Most qualitative studies (17/21) had a clear research question, with 95% (20/21) of articles describing results appropriately. The qualitative articles that were screened using the Critical Appraisal Skills Program method largely met the requirements. One criterion for consideration of ethical issues was met by one-third of the articles examined [35,37,50].

The quantitative articles, which were examined with the MMAT and the Cochrane Risk of Bias screening criteria, were able to fulfil the criteria across the board. Most quantitative articles had a low bias. The MMAT criteria were largely met.

For mixed methods articles, the quality of 1 [46] out of 11 articles could not be fully assessed because we considered the research question of this article to be unclear. The remaining mixed method articles satisfied the majority of the criteria (10/11, 91%). One article [79] did not show exact outcomes; thus, the criteria could not be fulfilled unambiguously.

## Discussion

### Principal Findings

In our mapping review, we found that older ’adults’ intention to use technology was driven by 6 different categories: Demographics and Health Status; Emotional Awareness and Needs; Knowledge, Competence, and Perception; Motivation; Social Influencers; and Actual Technology Features. These categories could be mapped to 2 different main technology types (everyday technologies and remote or assistive care technologies) and 3 purposes (health and safety, social interaction, and independence).

Our mapping review provides an overview of the application of existing theoretical models of technology acceptance while identifying additional key factors contributing to the intention to use digital technology among older adults. Most articles did not describe an existing technology adoption model. In the last 4 decades we found insufficient attention on research of technology adoption models specifically in the health sector, and more recent empirical research presenting major gaps in the actual application of theoretical models. Most articles lacked an explicit theoretical approach, which makes it difficult to interpret and compare the results of studies in this area.

New models incorporating constructs such as belief, resilience, and health status to facilitate the intention to use digital technology for older adults were found, alongside previous reports of individual influencing factors (eg, age, gender). Synonymous with previous reviews on the topic of technology acceptance and intention to use, our review suggests that despite 2 models being popularly used (TAM and UTAUT), older adults’ acceptance of technology was influenced by factors beyond the key constructs of these models.

Existing reviews have investigated older adults’ acceptance and intention to use technology across multiple phases of technology implementation [95-98]. Similar to the extrapolations of our mapping review, other reviews have found diversity in the variables influencing intention to use digital technology among older adults, with individual variables also being considered. This includes age (eg, older age); health status factors such as mobility issues (eg, fractured wrists and fingers) and vision discomfort; technology features; and support factors such as ongoing costs and accessibility of instructions and guidance [97]. A recent meta-analysis also highlighted that social influences (ie, conversations with family, friends, and professional caregivers) had a strong impact on the intention to use digital technology, especially when it is new and in the beginning of the adoption stages [96]. Factors including personality, beliefs, and resilience were found in our review, suggesting a dynamic influence of psychosocial traits on technology. These preferences and concerns adjust over the course of time and technology implementation [98,99].

There have been multiple studies exploring how existing technology models contribute to older adults’ intention to adopt technology. However, research on technology adoption and older people should go beyond describing facilitators and barriers [100]. Instead, a better understanding of the environment of older people and their interaction with this environment should be developed [100]. We believe that uncovering the role of communication and interaction between older people and their environment should be a key health research concern, as a means of contributing to improved care for older people in the community.

Taken together, the findings from this mapping review have important repercussions on the validity and applicability of popular theoretical models of technology adoption and intention to use digital technology. Our findings correspond with those of previous research expressing concern about the impact of unexplored factors along with their potential interaction with key components of commonly used technology adoption models and their subsequent reduced predictive ability [95]. We have used the findings of previous research along with the additional constructs found in our mapping review to suggest contributions to the discourse around technology adoption and intention to use digital technology specifically in relation to older adults.

### Implications for Research

Traditional models of technology adoption have been largely developed from information systems and health behavior models (eg, Table 3, Figure 2). However, factors from different disciplines have not been successfully combined so far. It has been shown that health-related and social-psychological factors also represent a major element in the adoption of technology, especially for older people. Our mapping review highlights that traditional information system models such as the TAM and UTAUT require the implementation of health and psychology constructs. Here, we identified a significant gap in the research. Furthermore, it is essential to note that existing models do not particularly focus on the age of technology users. The importance and awareness of the role of age in information systems were already highlighted by Tams et al [101]. They pointed out that future research needs to focus more extensively on cognitive age rather than on physical age, as older people show difficulties especially in dealing with complex and contemporary technologies. Perceived physical old age as opposed to cognitive old age, as well as perceived health status, constitutes further factors of investigation.

The advantage of this approach is that it emphasizes the relevant beliefs and antecedents for general intention to use digital technology and, consequently, provides more directive insights for the design of intention to use digital technology. Based on results, we underline the nature of personality (eg, attitude, self-efficacy expectation, resistance to change, resilience), emotions and social influences as well as the cognitive age, which are linked to health-promoting or health-damaging behavior and can have an activating effect on the intention to use digital technology [102,103]. Alternative factors including demographic and health data should be incorporated to ensure models can be applied appropriately (ie, at multiple stages of implementation), are flexible (ie, to account for different types of technology and changing older adult preferences), as well as being both individual- and context-dependent to assist with sustained intention to use digital technology (eg, inclusive of health status and disabilities). However, application of our framework should be considered in the context of the research objective and adapted accordingly. For example, while these constructs provide a basis for understanding early users’ intention to use digital technology by specifically focusing on the attitudinal, social, and normative belief structure, functional features of the technology type need to be considered for the design, implementation, and intention to use digital technologies (eg, ease of interoperability, size of screen), as well as the research question.

### Limitations and Further Research

Our review had a comprehensive search strategy covering social sciences, health care, and technology fields. However, it found a relatively small number of studies covering broad technology types and included limited theory-based studies as well as studies in conference proceedings. However, as ours is a mapping review, we focused mainly on identifying evidence-based gaps and trends across technology adoption in older adults more generally and the work was intended to be broad in scope. This may result in studies being overlooked (eg, from other databases and conference proceedings that are not peer-reviewed). Future studies should expand the scope to identify other factors and outcome variables, with more specific terms to capture wider forms of technology (eg, “digital assistants” or “bots”), as well as more inclusive eligibility criteria.

An additional limitation is that we discuss the stated theoretical models as equivalents without elaborating on their respective quality criteria. Previous research has already examined these models in the context of their research and shown variances, but there are considerably more opportunities to examine different age groups especially and to constantly include newly developed technologies. Future research could focus further on individual models in connection with specific categories of new and innovative technologies. Here, the focus should be on the health sector in particular. Existing studies were dominated by references to the TAM and UTAUT. Studies that pay more attention to interaction dimensions, emotional dimensions, and other resilience factors were lacking. Future research should bring these paradigms closer. While data on factors influencing use in the preadoption phase are extensive, findings on the postadoption phase were limited. To support the independence of community-dwelling older people over long periods, more research is needed to understand what influences the continued or sustained intention to use digital technologies after their introduction. Furthermore, additional quantitative research is required to understand which factors may have a greater impact than others and to examine the moderating or mediating relationships between factors.

### Conclusion

Technology acceptance is influenced by numerous factors. Existing models of technology acceptance should be more intensively integrated and revised. However, there is limited research on technology acceptance among older adults. Further research targeting understanding of the complexity and timing of the acceptance process of different types of technology by older adults is warranted.

## Appendix

Multimedia Appendix 1 Additional details on data extraction and quality appraisal.

## Abbreviations

mHealth: mobile health
MM: mixed method
MMAT: Mixed Methods Appraisal Tool
PRISMA: Preferred Reporting Items for Systematic Reviews and Meta-Analyses
TAM: Technology Acceptance Model
TAM2: Technology Acceptance Model 2
TAM3: Technology Acceptance Model 3
UTAUT: Unified Theory of Acceptance and Use of Technology
UTAUT 2: Unified Theory of Acceptance and Use of Technology Model 2
