# Supplementary material for: Older Adults and New Technology: Mapping Review of the Factors Associated With Older Adults’ Intention to Adopt Digital Technologies
Source: JMIR Aging. 2023 May 16;6:e44564. doi: 10.2196/44564 (PMC10230357; doi:10.2196/44564)
Supplement: Multimedia Appendix 1 [file aging_v6i1e44564_app1.docx]

**Supplementary Material**

*Data extraction*

A standardised form was used by the two reviewers to extract data from the selected studies, which is available as supplementary material. The data extraction form was developed specifically for this systematic review (based on other forms already developed/JBI data extraction forms for different study designs) to ensure all relevant data were collected. The first part of the extraction form includes entries on inclusion and exclusion criteria, quality assessment, methods used, type of technology studied and implementation stage. Articles were also checked for working definitions of acceptance and the use of existing technology acceptance models. This process was initially completed independently by the two reviewers who then conferred and combined their findings. Both reviewers became familiar with the standardised form prior to data extraction to ensure consistency throughout the process. As articles under review used either qualitative methods, quantitative methods or a combination of both (mixed methods), to extract factors from all types of articles, the data extraction form contained a section for extracted factors based on the type of data (qualitative vs quantitative). In the case of qualitative articles and qualitative data from mixed methods articles, factor names and their perceived influence on acceptance and intention to use technology were coded and subsequently entered in the qualitative section of the form. In the case of quantitative articles and quantitative data from mixed methods articles, the following information was entered: variable name, standardized or unstandardized regression coefficients, level of significance, and proportion of variance explained. If studies were identified to have missing data, the corresponding author was contacted to clarify the information required.

*Search Strategy*

An initial search will be conducted on EMBASE to highlight potential studies using the following combination of four groups of keywords:

- aged, older, senior, elderly,
- acceptance, adoption, use, adherence, rejection
- behaviour, psychology, motivation
- technology, computers, eHealth, system, assistive technology, robotics, smart home, gerontechnology, telemonitoring

The titles and abstracts of studies located in this search will be reviewed for keywords and common text to help build the search strategy for this systematic review. A detailed search will then be conducted using the identified keywords across several databases:

1. Psychology / Health Informatics / Gerontology
2. CINAHL Complete, MEDLINE Complete, PubMed, EMBASE, and Scopus
3. Information Systems
4. AIS Electronic Library, IEEExplore, Senior Scholar Basket Journals

*Tables*

[Table 1. Quality Appraisal with CASP for qualitative studies 2](#_Toc128589491)

[Table 2. Quality Appraisal with Cochrane risk of bias for quantitative studies 2](#_Toc128589492)

[Table 3. Quality Appraisal with MMAT for mixed methods 3](#_Toc128589493)

[Table 4. Quality Appraisal with MMAT - Updated search for conference paper 3](file:///C:\Users\46076409\Dropbox\Joint%20Senior%20Research\JMIR%20SR%20Tech%20Adoption%20Supplementary%20Material_20230301.docx#_Toc128589494)

Table 1. Quality Appraisal with CASP for qualitative studies


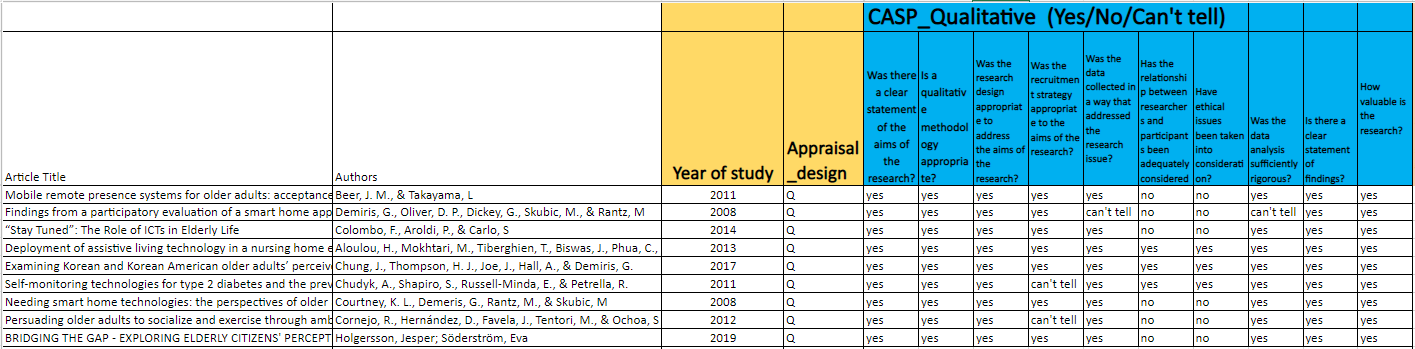


Table 2. Quality Appraisal with Cochrane risk of bias for quantitative studies


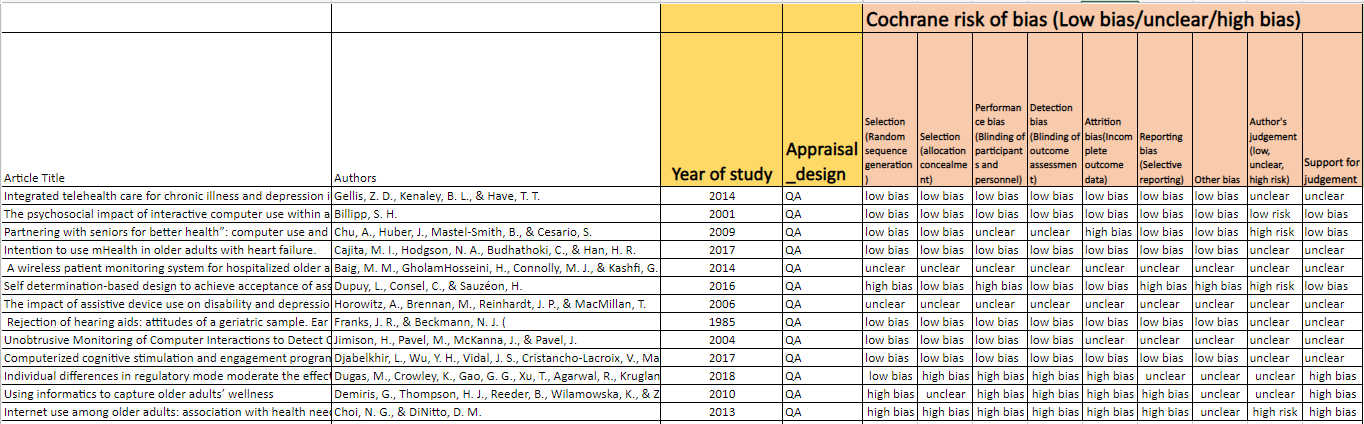


Table 3. Quality Appraisal with MMAT for mixed methods


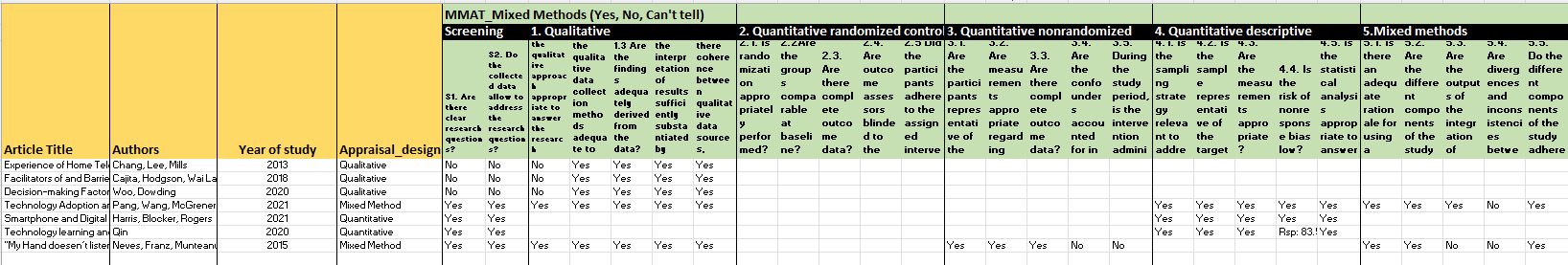

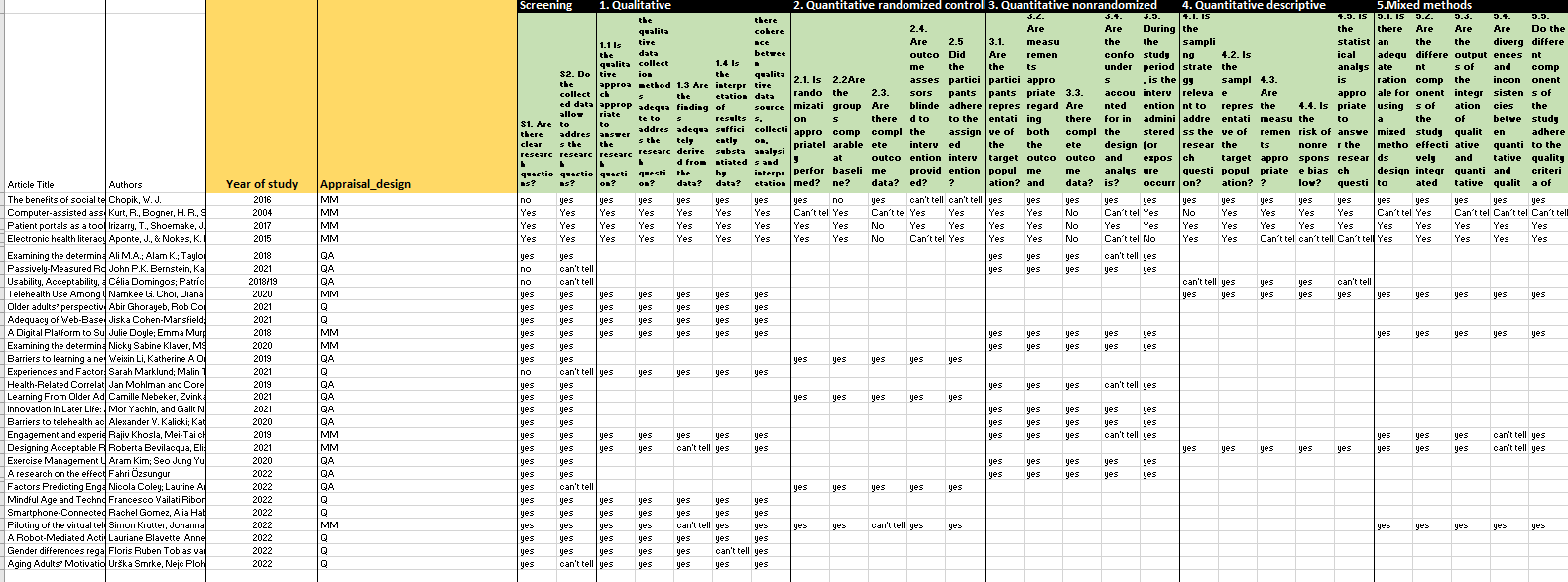


Table 4. Quality Appraisal with MMAT - Updated search for conference paper
